# Urinary incontinence following subtotal and total hysterectomy: a systematic review

**DOI:** 10.31744/einstein_journal/2019RW4320

**Published:** 2019-04-22

**Authors:** Priscila Scalabrin Longo, Laura Virilo Borbily, Felipe Placco Araujo Glina

**Affiliations:** 1 Universidade de Santo Amaro, São Paulo, SP, Brazil; 2 Department of General Surgery, Faculdade de Medicina de Jundiaí, Jundiaí, SP, Brazil

**Keywords:** Hysterectomy, Uterine diseases, Urinary incontinence, Histerectomia, Doenças uterinas, Incontinência urinária

## Abstract

**Objective:**

To evaluate the best surgical approach for the female urinary incontinence.

**Methods:**

Systematic review conducted in MEDLINE^®^ Cochrane, EMBASE and LILACS database up to September 1^st^, 2017. Articles were selected according to study type, type of intervention and outcomes. Articles were selected by more than one researcher based on title, abstract and full text. The SIGN checklist was used for bias assessment.

**Results:**

A total of 165 articles were retrieved from MEDLINE^®^ . Twenty-five studies were elected for full text reading, and 11 of them were selected for the final text analysis. The heterogeneity between questionnaires used in different studies precluded a meta-analysis of results.

**Conclusion:**

This study yielded evidences supporting the hypothesis that total and subtotal hysterectomy have different impacts on urinary function of patients with benign uterine diseases. Articles revealed higher frequency of urinary incontinence following subtotal compared to total hysterectomy.

## RESUMO

**Objetivo:**

Avaliar qual a melhor conduta cirúrgica na incontinência urinária feminina.

**Métodos:**

Revisão sistemática conduzida no MEDLINE^®^, Cochrane, EMBASE e LILACS até 1º de setembro de 2017. Os artigos foram escolhidos de acordo com o tipo de estudo e de intervenção, e seus resultados. Os artigos foram selecionados por mais de um pesquisador, com base no título, no resumo e no texto completo. O *checklist* SIGN foi usado para avaliar vieses.

**Resultados:**

No MEDLINE^®^, foram recuperados 165 artigos. Foram escolhidos 25 estudos para leitura do texto completo, e somente 11 foram escolhidos para análise do texto final. Não foi realizada metanálise dos resultados devido à heterogeneidade dos questionários usados em cada estudo.

**Conclusão:**

Encontrou-se evidência que suporta a hipótese de que as histerectomias total e subtotal causam impactos diferentes na função urinária de pacientes com doença uterina benigna. Os artigos mostraram maior frequência de incontinência urinária após histerectomia subtotal quando comparada à total.

## INTRODUCTION

Hysterectomy is widely performed among American patients, and therefore draws more attention.^(^ ^1^ ^)^ As a major gynecological surgery in the United States, the procedure implies greater concern with patient satisfaction compared to other alternative treatments for benign uterine diseases.^(^ ^2^ ^)^ Fibroids, abnormal premenopausal or postmenopausal uterine bleeding, endometriosis, chronic pelvic pain and pelvic organ prolapse are common conditions^(^ ^3^ ^)^ among women at different ages.

Subtotal abdominal hysterectomy (SAH) is thought to be the procedure of choice for patients with benign uterine diseases; however, recent studies have shown that total abdominal hysterectomy (TAH) may be beneficial^(^ ^3^ ^–^ ^6^ ^)^ regarding post-operative complications, with decreased operative morbidity^(^ ^7^ ^)^ and reduced risk of urinary and sexual dysfunction. In the United States, the number of subtotal hysterectomies has increased.^(^ ^8^ ^)^

Variables derived from intra-operative and post-operative events and complications among women undergoing SAH or TAH include duration of operation, blood loss, length of hospital stay, hemoglobin values, pain score, readmission, urinary retention, surgical wound infection, ileus, vaginal bleeding, bowel obstruction, cervical prolapse, persistent pain and other factors.^(^ ^6^ ^,^ ^9^ ^)^

In patients with benign uterine disease, urinary incontinence may result from medical intervention, with different rates between patients submitted to TAH or SAH.^(^ ^10^ ^)^

This study was designed to compare urinary incontinence as a primary outcome of SAH or TAH for treatment of benign uterine diseases.

## METHODS

### Inclusion and exclusion criteria

Selected articles were randomized clinical trials published in English, Portuguese or Spanish, which included women with benign uterine diseases. Interventions consisted of total hysterectomies via abdominal or vaginal access, and were compared to subtotal hysterectomies by the same approaches. Urinary function was the selected outcome, measured by validated questionnaires.

Studies involving malignant diseases, different interventions, different comparisons, outcomes other than urinary dysfunction and use of non-validated questionnaires were excluded.

### Databases

Articles were retrieved from MEDLINE^®^ via PubMed, Cochrane, *Literatura Latino-Americana e do Caribe em Ciências da Saúde* (LILACS) and EMBASE search until/on September 1^st^, 2017. The following search strategy was used: “total hysterectomy AND subtotal hysterectomy AND (urinary incontinence) AND benign uterine disease”.

### Selection

#### Selection process

Eligibility assessment was performed independently by two reviewers, in a non-blinded, standardized fashion. Disagreements between reviewers were resolved by consensus. Studies were considered at each stage (title, abstract and full text) of the process for the sake of better selection. Study authors were not contacted.

#### Checklist

The Scottish Intercollegiate Guidelines Network (SIGN)^(^ ^11^ ^)^ checklist was used to evaluate clinical trials.

### Critical evaluation

#### Biases

Selection, performance, detection, misunderstanding and reporting were considered biases.

To ascertain the validity of eligible clinical trials, independent, reliable peer reviewers determined the adequacy of allocation concealment and blinding of patients, health care providers, data collectors and outcome assessors. All items above were contemplated in the SIGN evaluation questionnaire.

#### Extraction results

Results were selected from all articles evaluating urinary incontinence via validated questionnaires.

## RESULTS

### Study selection

PubMed, Cochrane, LILACS and EMBASE database search yielded 640 records in MEDLINE^®^ and in other databases, with 630 records remaining after excluding duplicates. Of these, 165 records were screened, 25 full-text articles assessed for eligibility and 9 studies included in qualitative synthesis. Four articles were excluded for not describing clinical trials. One full-text article published in Hebrew and one in Italian, three articles that did not contain separate analysis of the intervention or comparison of results, and two articles including patients with malignant diseases were also excluded.

**Figure 1:**
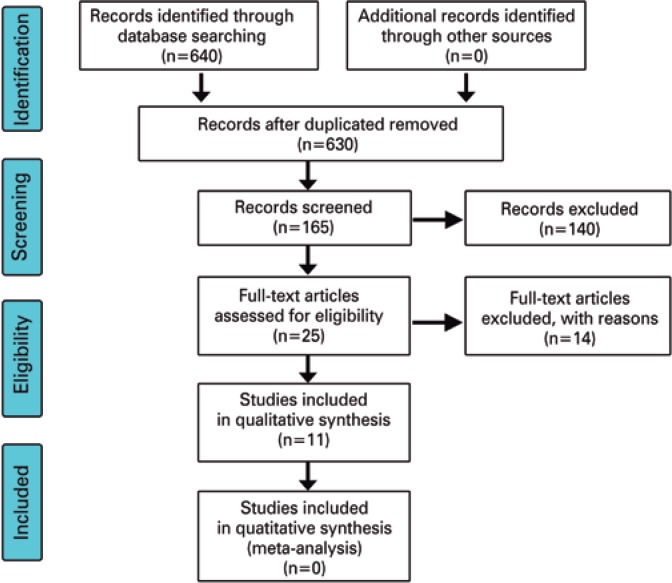
Flow of information through the different phases of a systematic review

A total of 25 articles were retrieved, excluding textbooks and dissertations. The search and selection strategy employed was displayed in the PRISMA^(^ ^12^ ^)^ flowchart (Figure 1); the exclusion reasons for articles are shown in table 1.

**Table 1:** Reasons for exclusion of articles retrieved

Article	Reasons for exclusion
Kocaay et al.^(^ ^1^ ^)^	Does not contain separate analysis of the intervention or comparison of results
Ala-Nissilä et al.^(^ ^2^ ^)^	Not randomized
Selcuk et al.^(^ ^8^ ^)^	Includes patients with malignant diseases
Gilbaz et al.^(^ ^9^ ^)^	Includes patients with malignant diseases
Rabinerson et al.^(^ ^10^ ^)^	Full text in Hebrew
Al-Mehaisen et al.^(^ ^13^ ^)^	Does not contain separate analysis of the intervention or comparison of results
Morelli et al.^(^ ^14^ ^)^	Full text in Italian
Engh et al.^(^ ^15^ ^)^	Not a clinical trial
Walsh et al.^(^ ^16^ ^)^	Not a clinical trial
El-Toukhy et al.^(^ ^17^ ^)^	Not a clinical trial
Neumann et al.^(^ ^18^ ^)^	Does not contain separate analysis of the intervention or comparison of results
Roovers et al.^(^ ^19^ ^)^	Not a clinical trial
Gimbel et al.^(^ ^20^ ^)^	Not randomized
Gimbel et al.^(^ ^21^ ^)^	Crossover of 22%

### Study characteristics

All nine studies selected for review were randomized controlled trials published in English or Portuguese. Articles can be found in table 2, along with descriptions of sample size, follow-up time, type of access, type of study, type of questionnaire and patient characteristics.

**Table 2:** Study description

Articles	Study type	Patients	TH	STH	Questionnaire	Follow up (years)	Number of participants (intervention/ comparison group)
Andersen et al.^(^ ^3^ ^)^	RCT	ND/BUP	A	A	PFDI-20	14	158/161
Andersen et al.^(^ ^4^ ^)^	RCT	BUP, >18 years	A	A	PFDI-20/SF-36	14	100/97
Andersen et al.^(^ ^5^ ^)^	RCT	BUP, >18 years	A	A	PFDI-20/SF-36	5	158/161
Greer et al.^(^ ^6^ ^)^	RCT	BUP, PREM 30-50 years	A	A	HRQOL/SF-36	9	27/27
Andersen et al.^(^ ^7^ ^)^	RCT	ND/BUP	A	A	PFDI-20/SF-36	14	100/97
Gimbel et al.^(^ ^20^ ^)^	RCT	BUP	A	A	IIQ	0 and 1	158/161
Learman et al.^(^ ^22^ ^)^	RCT	BUP	A	A	IIQ	2	68/67
Thakar et al.^(^ ^23^ ^)^	RCT	BP	A	A	IIQ	1	133/146

* TH: total hysterectomy; STH: subtotal hysterectomy; RCT: randomized clinical trial; ND: not described; BUP: benign uterine pathology; BP: benign pathologies; PFDI-20: Pelvic Floor Disability Index; SF-36: 36-Item Short Form Health Survey; PREM: premenopausal; HRQOL: Health Related Quality of Life; IIQ: Incontinence Impact Questionnaire.

#### Risk of bias within studies

Potential study biases are shown in table 3. The SIGN^(^ ^11^ ^)^ checklist was used to assess methodological quality and data reliability in selected studies.

**Table 3:** The Scottish Intercollegiate Guidelines Network (SIGN) checklist^(^ ^12^ ^)^

SIGN checklist	Andersen et al.^(^ ^3^ ^,^ ^4^ ^,^ ^7^ ^)^	Andersen et a.^(^ ^5^ ^)^	Greer et al.^(^ ^6^ ^)^	Gimbel et al.^(^ ^20^ ^)^	Gimbel et al.^(^ ^21^ ^)^	Learman et al.^(^ ^22^ ^)^	Thakar et al.^(^ ^23^ ^)^
1.1. The study addresses an appropriate and clearly focused question	Yes	Yes	Yes	Yes	Yes	Yes	Yes
1.2. The assignment of subjects to treatment groups is randomized	Yes	Yes	Yes	No	Yes	Yes	Yes
1.3. Adequate concealment method is used	Cannot say	Yes	Yes	Cannot say	Yes	Yes	Yes
1.4. Subjects and investigators are kept “blinded” to treatment allocation	No	Cannot say	Yes	Cannot say	No	Yes	Yes
1.5. Treatment and control groups are similar at the start of the trial	Yes	Yes	Yes	Yes	Yes	Yes	Yes
1.6. The only difference between groups is the treatment being investigated	Yes	Yes	No	Yes	Yes	Yes	Yes
1.7. Relevant outcomes are measured in a standardized, valid and reliable way	Yes	Yes	Yes	Yes	Yes	Yes	Yes
1.8. What percentage of individuals or clusters recruited into each treatment arm of the study dropped out before study completion?	15%	Not described	Not described	15%	22.9%	Not described	Not described
1.9. All studies are analyzed in the groups to which they were randomly allocated (often referred to as intention to treat analysis)	Yes	Yes	Yes	Cannot say	Yes	Yes	Cannot say
1.10. For studies carried out in more than one site, results are comparable for all sites	Yes	Yes	Cannot say	Yes	Yes	Cannot say	Cannot say
2.1. How well was the study performed to minimize bias?	Acceptable	Acceptable	Acceptable	Acceptable	Acceptable	Acceptable	Acceptable

#### Results of individual studies

Andersen et al.,^(^ ^3^ ^)^ is a 14-year follow-up study describing an objective comparison of SAH and TAH with respect to pelvic organ prolapse and urinary incontinence. It is a randomized controlled trial of patients with benign indications − 319 women undergoing SAH (n=53) or TAH (n=47) abdominal hysterectomy. Urinary incontinence findings were as follows (for SAH and TAH, respectively): objectively assessed urinary incontinence (pad weighing test) − 26.4% and 29.8%; subjectively assessed urinary incontinence (questionnaire) − 39.6% and 34.04%; combined subjectively and objectively assessed urinary incontinence − 16.98% and 14.89%.

In another study, Andersen et al.,^(^ ^4^ ^)^ compared lower urinary tract symptoms following SAH and TAH via exploratory analysis of a 14-year follow-up randomized clinical trial. A total of 197 patients undergoing SAH (n=97) or TAH (n=100) hysterectomy for benign uterine diseases answered the questionnaire. Stress incontinence and constant/frequent urinary incontinence were reported in 62.5% and 45%, as well as 33% and 20% of cases (SAH and TAH, respectively).

Another study by Andersen et al.,^(^ ^7^ ^)^ compared SAH and TAH in a randomized clinical trial with 14-year questionnaire-based follow-up. Women referred for benign uterine diseases who did not have contraindications to SAH were randomized to SAH (n=161) or SAT (n=158) abdominal hysterectomy. The questionnaire was answered by 197 of 304 women. Urinary incontinence occurred in 33% and 20% of cases (SAH and TAH, respectively). A second study by the same authors^(^ ^7^ ^)^ reported on the 5-year follow-up of a randomized controlled trial comparing SAH with TAH, with 319 women referred with benign uterine diseases referred to abdominal hysterectomy (SAH, n=161; TAH, n=158). Only 234 women answered the questionnaire. Urinary incontinence occurred in 30.1% of women undergoing SAH and 17.6% of those submitted to TAH. Greer et al.,^(^ ^6^ ^)^ showed the long-term outcomes of the TOSH (Total or Supracervical Hysterectomy) trial in a 9-year study. A total of 54 patients (TAH, 27; SAH, 27) were included and only 37 women answered the questionnaire (19 and 18 for TAH and SAH, respectively). The study revealed itself in 45% and 27% of women submitted to TAH and 52% and 49% of those undergoing SAH considering baseline and long-term outcome, respectively. Urge incontinence was reported by 47% and 32% of women submitted to TAH, and 40% e 32% of women undergoing SAH (baseline and long-term outcome respectively).

In a 1-year follow-up study by Gimbel et al.,^(^ ^20^ ^)^ the incidence of urinary incontinence after TAH (10%) was lower than after SAH (19%). Greater decrease in the number of women with stress urinary incontinence and mixed urinary incontinence was noted in the TAH as compared to the SAH group. The number of women with urge urinary incontinence increased in the SAH group and decreased in the TAH group. Urinary incontinence was correlated with the item “lower urinary tract symptoms – interfering with her daily life”, in all 12 women in TAH the group and the 23 women in the SAH group.

Another study by Gimbel et al.,^(^ ^21^ ^)^ with 1-year follow-up, evaluated urinary incontinence, postoperative complications, quality of life (36-Item Short Form Health Survey − SF-36), occurrence of constipation and prolapse, satisfaction with sex life, and pelvic pain within 1 year of surgery. The percentage of patients with urinary incontinence 1 year after surgery was higher in the TAH group (15% and 10%, TAH and SAH, respectively). Patients in the TAH group also had more intra-abdominal abscesses/hematomas compared to the SAH group (7% and 1%, respectively). Pulmonary embolism (TAH, 0%; SAH, 2%), urinary tract infections (TAH, 1%; SAH, 6%) and abdominal pain (TAH, 3%; SAH, 8%) were more common among SAH compared to TAH patients. There was a statistically significant improvement in quality of life after surgery (physical score, p=0.0001; mental score, p=0.0001), regardless of surgical approach. Fourteen women (15%) submitted to SAH had persistent vaginal bleeding following hysterectomy. Three women in the SAH group had their cervix removed after hysterectomy: one due to intolerable vaginal bleeding, one due to continuous pelvic pain, and one due to leiomyosarcoma diagnosis in postoperative pathologic examination of the uterus.

Another study by Gimbel et al.,^(^ ^21^ ^)^ with 1-year follow-up revealed a significantly smaller proportion of women with urinary incontinence 1 year after TAH, as compared to SAH (9% and 18%, respectively). The lower proportion of women with incontinence in the TAH group reflected the higher proportion of symptom relief (TAH, 14%; SAH, 10%) as well as a lower proportion of women with new symptoms (TAH, 0.02%; SAH, 0.07%).

A randomized comparison conducted by Learman et al.,^(^ ^22^ ^)^ showed the different proportions of surgical complications and clinical outcomes 2 years after randomization. Patients assigned to SAH had more hospital readmissions than those assigned to TAH. The following urinary symptoms were considered (SAH and TAH, respectively): urinary urgency (8.2% and 9.4%), sensation of incomplete bladder emptying (6.6% and 1.6%), frequent urination (16% and 14%), stress incontinence (13% and 4.7%) and urge incontinence (6.6% and 3.1%). Overall, TAH was associated with less complications regarding urinary symptoms.

A 1-year follow-up study by Thakar et al.,^(^ ^23^ ^)^ revealed rates of urinary frequency of 32.8% and 31.4%, before SAH and TAH, and lower percentages within 12 months of surgery (23.5% and 19.8%, SAH and TAH, respectively), with more marked decrease in the TAH group. The reduction in nocturia and stress incontinence and the improvement in bladder capacity were similar in both groups. The frequency of bowel symptoms (estimated based on reported constipation and use of laxatives) and measures of sexual function (including frequency of intercourse and orgasm and rating of the sexual relationship with a partner) did not change significantly after surgery in either group. Women in the subtotal hysterectomy group had shorter length of hospital stay (5.2 compared to 6.0 days in the total hysterectomy group) and a lower incidence of fever (6% *versus* 19%).

## DISCUSSION

### Summary of evidence

The hypothesis that SAH would have a better impact on postoperative urinary incontinence occurrence compared to TAH was confirmed by literature data, which offered high quality, robust evidence revealing great improvement in the total hysterectomy approach. Only randomized clinical trials were included in this study. Small as the sample may be, follow-ups were long enough to show that results did not differ over time.

It was believed that TAH would imply higher rates of urinary incontinence compared to SAH. However, the selected studies revealed otherwise, as reported by Andersen et al.,^(^ ^3^ ^,^ ^7^ ^)^ Greer et al.,^(^ ^6^ ^)^ Learman et al.,^(^ ^22^ ^)^ and Thakar et al.^(^ ^23^ ^)^

Statistically significant aspects were highlighted in the studies of Andersen et al.,^(^ ^3^ ^,^ ^4^ ^,^ ^7^ ^)^ with occurrence of urinary incontinence in 33% of patients undergoing SAH, as compared to 20% of patients submitted to TAH, over a 14-year follow-up, and 30.1% and 17.6% of patients (SAH and TAH, respectively) over a 5-year follow-up.

As regards follow-up time, studies evaluated urinary disorders within 1 year of surgery. This may have been a major factor to explain heterogeneity among articles.

The 1-year follow-up study conducted by Gimbel et al.,^(^ ^21^ ^)^ addressed not only urinary incontinence, but also postoperative complications, quality of life (SF-36), occurrence of constipation and prolapse, satisfaction with sex life and pelvic pain within one year of surgery.

The 1-year follow-up study of Thakar et al.,^(^ ^23^ ^)^ revealed similar reduction in nocturia and stress incontinence, and improvement in bladder capacity in both groups. Frequency of bowel symptoms, estimated based on reported constipation and use of laxatives, and measures of sexual function, including the frequency of intercourse and orgasm and rating of sexual relationships with a partner, did not change significantly after surgery in either group.

Articles reporting data derived from well-designed questionnaires were the focus and core of this review, given the high levels of dependence, standardization and reliability. The Incontinence Impact Questionnaire (IIQ) deserved attention due to its high capacity to collect and converse the pieces of information studied. The Health Related Quality of life (HRQOL) was also thought to be important, mainly due to the organization method employed.

### Limitations

Besides prevailing outcomes in this systematic review, some particularities of selected studies should be accounted for. Some factors, such as size of the sample population, loss to follow-up, surgical approach employed, and outcomes evaluated are vital for proper understanding of the results presented.

The small sample size is a potential limitation in Gimbel et al.,^(^ ^20^ ^)^ and Thakar et al.^(^ ^23^ ^)^ Significant loss to follow-up in Andersen et al.,^(^ ^3^ ^)^ and Andersen et al.,^(^ ^4^ ^)^ was regarded as a potential bias.

Another key aspect is the surgical approach employed in different studies. Total abdominal hysterectomy and SAH were performed exclusively via the abdominal approach in 11 studies. The laparoscopic approach was not used; however, there is no consensus in the literature regarding the best surgical approach. Comparison of surgical procedures based on different approaches may have introduced a bias and impacted reported results. The vaginal approach is also commonly used in total hysterectomy.

The heterogeneity of results derived from the use of different questionnaires precluded conduction of a meta-analysis. Much discrepancy among the studies favored the option for a systematic review, even though this may be a negative point as far as the literature is concerned.

The use of a standard and validated questionnaire would be ideal to promote homogeneity of urinary outcomes in literature analysis. However, no single model exists for comprehensive analysis encompassing all outcomes, and different questionnaires are used in most studies.

## CONCLUSION

There is evidence to support the hypothesis that total hysterectomy has less impact on urinary function as compared to subtotal hysterectomy. Still, larger multicenter studies based on data derived from validated questionnaires are warranted to further support the evidence presented.
